# The climatic association of population divergence and future extinction risk of *Solanum pimpinellifolium*

**DOI:** 10.1093/aobpla/plaa012

**Published:** 2020-03-12

**Authors:** Ya-Ping Lin, Cheng-Yueh Lu, Cheng-Ruei Lee

**Affiliations:** 1 Institute of Ecology and Evolutionary Biology, National Taiwan University, Taipei, Taiwan; 2 Institute of Plant Biology, National Taiwan University, Taipei, Taiwan; 3 Genome and Systems Biology Degree Program, National Taiwan University, Taipei, Taiwan

**Keywords:** climate change, isolation by environment, *Solanum pimpinellifolium*, species distribution modelling

## Abstract

Under intraspecific differentiation driven by differential climatic adaptation, it may be expected that intraspecific genetic groups occur at distinct environments. Populations occupying different niches may therefore differ in their ability to cope with climate change. Here, we addressed this hypothesis with a wild tomato, *Solanum pimpinellifolium*. This species is distributed from the west side of Andes to the coastal region in Peru and Ecuador and occupies a wide environmental diversity. This environmental diversity is related to the genetic structure of the species providing an ideal material to investigate the isolation by environment hypothesis. While previous hypothesis stated that *S. pimpinellifolium* originated from northern Peru and migrated northwards and southwards, our results support that *S. pimpinellifolium* originated from Ecuador and expanded to northern and southern Peru, and during this process, the niche space of *S. pimpinellifolium* became more associated with cold and drought. We further predicted its fate under anthropogenic climate change. According to our predictions, the northern group will maintain its current extent or even expand to the entire western region of Ecuador. In contrast, we predicted low habitat suitability for the southern group which could potentially lead to the shrinkage of its distribution. In conclusion, we revealed the distinct fates among the differentiated populations driven by environment under global warming conditions.

## Teaser

We proposed a hypothesis that *Solanum pimpinellifolium* originated from Ecuador and diverged southwards into genetically differentiated groups in Peru, and during the divergence, the niche became more associated with drought and cold. We showed that the ancestral group in Ecuador may maintain or even expand its distribution while the group in southern Peru could shrink its distribution in scenarios with the worst global warming, suggesting distinct future fates among genetic groups may be driven by their unique environmental adaptation.

## Introduction

Genetic differentiation among populations could be affected by neutral forces as well as differential local adaptation. Due to the limitation of dispersal, genetic drift would make species diverged into distinct populations, so-called isolation by distance, and increase the genetic distance among geographically distant populations (Wright 1943; Slatkin 1987; Hutchison and Templeton 1999; Clegg and Phillimore 2010). Alternatively, when populations are locally adapted to distinct environments, the maladaptation of immigrant or hybrid individuals with the local environment can reduce effective gene flow and enhance genetic differentiation among populations. Under such ‘isolation by environment’, strong correlation between genetic and environmental distances after controlling for geographic distance may be expected (Sexton *et al.* 2014). Indeed, researches have revealed that differential local adaptation exists and contributes to the genetic variation within species (Zuriaga *et al.* 2009; Lee and Mitchell-Olds 2011; Fang *et al.* 2014; Yoder *et al.* 2014).

Anthropogenic climate change affects the species distribution generally in a way of reducing suitability of a species in its original range or even diminishing its distribution (Alsos *et al.* 2012; Gugger *et al.* 2018; Exposito-Alonso *et al.* 2019). To model the potential distribution change, species distribution modelling is often employed (Guisan and Thuiller 2005; Alvarado-Serrano and Knowles 2014). However, classical species distribution modelling such as MaxEnt assumes a species is a homogeneous group having identical niche space (Davis and Shaw 2001; Atkins and Travis 2010; Banta *et al.* 2012; Hällfors *et al.* 2016). Given the undeniable evidence of differential local adaptation of diverse populations within species, such assumption in standard species distribution modelling might be misleading. Indeed, recent studies have emphasized and found solid evidence that locally adapted populations within species would respond to climate change differently. In these studies, researchers first separated samples within a species into several genetic groups and performed species distribution modelling separately for each group (Ikeda *et al.* 2017; Bay *et al.* 2018; Razgour *et al.* 2019). Here, we used another complementary method to address the heterogeneous genetic variation in species distribution modelling.

To connect the relationship between genetic variation and environment, we first identified the adaptive loci of environmental variables and used gradient forest analysis to investigate the association between adaptive loci along the environmental gradients (Ellis *et al.* 2012). This association not only determines the importance of environmental variables but also detects a response threshold where the major change of genetic variation occurs. An extension of gradient forest analysis is genetic offset that measures the distance between current and future genome–environment association (Fitzpatrick and Keller 2015). High genetic offset indicates the current genetic composition of a population is maladaptive to future environments (Fitzpatrick and Keller 2015; Gugger *et al.* 2018; Keller *et al.* 2018). Through the integration of genetic variation with species distribution modelling, we expect to discover the environmental variables that contribute to local adaptation and to reveal the fates of locally adaptive populations under global climate change.

An ideal material to investigate environment-driven population differentiation is wild tomato. Tomato clade occupies a diverse range of habitats along a wide climatic gradient, and also, some of the wild tomatoes present intraspecific population differentiation (Moyle 2008; Böndel *et al.* 2015; Lin *et al.* 2019). Among several wild tomatoes, *Solanum pimpinellifolium* particularly draws our attention because it is a close relative of cultivated tomato, *Solanum lycopersicum* (Bedinger *et al.* 2011). *Solanum pimpinellifolium* is native in Ecuador and Peru, along the west side of Andes to the coastal region (Rick *et al.* 1977). From north to south, the climate in the region ranges from tropical rainforest to cold arid desert (Kottek *et al.* 2006). Previous studies suggested that northern Peru to be the centre of origin of *S. pimpinellifolium* because accessions in northern Peru showed higher genetic diversity than northernmost (Ecuador) and southernmost (southern Peru) (Rick *et al.* 1977; Zuriaga *et al.* 2009; Blanca *et al.* 2015; Lin *et al.* 2019). To address the population–environment interaction, it is necessary to define the genetic groups and their ecological niches. However, unlike the cultivated tomato, *S. pimpinellifolium* presents high genetic diversity and its population structure could not be identified properly using genome-wide simple sequence repeat markers (Zuriaga *et al.* 2009; Rao *et al.* 2012). Genetic groups of this species remained unclear until a recent study using genome-wide high-density single nucleotide polymorphism (SNP) markers (Lin *et al.* 2019). Three populations with single ancestry clustered in Ecuador, northern Peru and southern Peru while populations derived from two independent ancestors were located intermediately between their donated populations (Lin *et al.* 2019). Given that the population differentiation of *S. pimpinellifolium* is associated with climatic conditions (Zuriaga *et al.* 2009; Blanca *et al.* 2015; Gibson and Moyle 2020), we expect these three single-ancestral populations differ in their ecological niches.

In this study, we started from a phylogenetic tree to infer the population divergence. Afterwards, species distribution modelling was achieved to identify the important bioclimatic variables that defined these niches. We also predicted the future species distribution by 12 scenarios of global climate models, with and without the integration of genome–environment association, to investigate the extinction risk of *S. pimpinellifolium*.

## Materials and Methods

### Locations, bioclimatic variables and sequences

The 94 accessions of *S. pimpinellifolium* were originally requested from Tomato Genetics Resource Center, UC Davis. We re-classified these 94 accessions according to the posterior probability of ADMIXTURE in a previous study (Lin *et al.* 2019). If an accession has posterior probability belonging to one genetic group > 0.9, it was classified as single ancestral (**see****Supporting Information—Table S1**, modified from Lin *et al.* (2019)). The other accessions are classified as admixtures. The geographic location and the sequences were downloaded from the previous study (Lin *et al.* 2019). For the downloaded RAD SNP data set, we removed SNP with heterozygosity > 3 times of standard deviation of all SNP sites in the following analyses, resulting in about 20 000 SNP. The minimum, maximum, mean temperature and precipitation per month of each accession were downloaded from WorldClim 1.4 (Hijmans *et al.* 2005).

### Phylogenetic analysis

We used all RAD sequencing sites to make a maximum likelihood phylogenetic inference (Stamatakis 2014). To obtain all the sites, GATK3.7 was executed following the manual (McKenna *et al.* 2010; Depristo *et al.* 2011; Van der Auwera *et al.* 2013). We removed sites in a condition of missing value > 10 %, heterozygosity > 3 times of standard deviation of all sites, read depth < 10 and read depth > 3 times of standard derivation of all sites, resulting in about 3.3 million sequencing sites including non-variant sites across all samples. The outgroup was tomato reference genome SL2.50 (Heinz 1706), which belonged to *S. lycopersicum* (The Tomato Genome Consortium 2012). The maximum likelihood phylogenetic analysis was performed using RAxML following the manual (Stamatakis 2014). The parameters were set to 20 maximum likelihood searches on 20 randomized stepwise addition parsimony trees and 100 bootstraps. To investigate the genetic diversity of these populations, we calculated the mean pairwise nucleotide polymorphism (π) using the 3.3 million sequencing sites by TASSEL (Bradbury *et al.* 2007).

### Species distribution modelling

**Figure 1.**
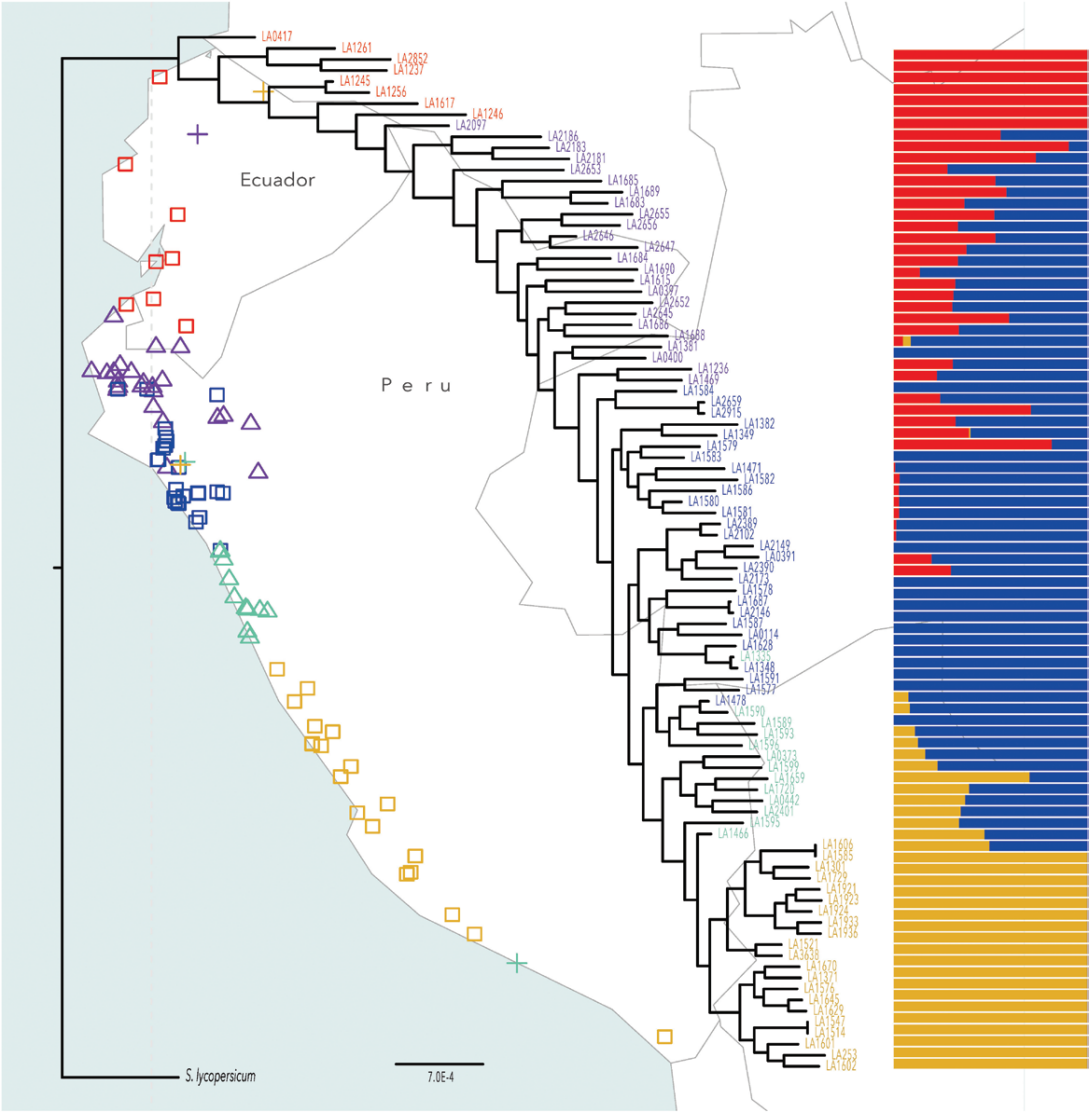
Phylogenetic tree and the location of *S. pimpinellifolium* using *S. lycopersicum* as outgroup. The red labels indicate the northern population; blue for the central population; goldenrod for the southern population; purple for centre-north population (admixture between the northern population and the central population); aquamarine for centre-south population (admixture between the central population and the southern population). The + indicates the accessions which are geographically distant from all other accessions from the same genetic cluster and therefore removed from further analyses, including LA1236, LA1335, LA1466, LA1547 and LA1585. The bars on the right side are the result of ADMIXTURE and ordered by the accessions latitude from north to south (top to bottom).

The first objective was to investigate the niches of these populations since the genetic distance of *S. pimpinellifolium* was correlated to the climatic distance (Zuriaga *et al.* 2009). Before species distribution modelling, five accessions were excluded, including LA1236, LA1335, LA1466, LA1547, LA1585, because they were geographically distant from all other accessions of the same genetic cluster (Fig. 1), likely resulting from mis-recording of their locations or sample contamination. To prepare the bioclimatic variables, 30 arc-second resolution of the current climate data (average for 1960–90) were downloaded (Hijmans *et al.* 2005). Since empirical studies have shown that the prediction of species niche may differ among general circulation models, future climate information of CCSM4 (CC), HadGEM2-AO (HD) and Miroc-ESM (MR) were used to avoid misleading by single general circulation model (Beaumont *et al.* 2008; Gent *et al.* 2011; Martin *et al.* 2011; Watanabe *et al.* 2011; Goberville *et al.* 2015). We prepared the data of 30 arc-seconds resolution for 2050 (average for 2041–60) and 2070 (average for 2061–80) with two greenhouse gas emission scenarios, representative concentration pathway (RCP) 2.6 and RCP 8.5. Representative concentration pathway 2.6 assumes global greenhouse gas emission will be under control and decline substantially in the future. Representative concentration pathway 8.5 simulates that the global greenhouse gas emission will grow continually in the worst condition. We extracted all the bioclimatic variables in a frame ranging from *x*-axis: −83.03070 to −69.324503 and *y*-axis: −19.11173 to 2.270769, which contained all the collection sites using R package raster (Hijmans 2019). In addition, highly correlated bioclimatic variables were removed (correlation > 0.9), resulting in nine bioclimatic variables in this study. The nine bioclimatic variables included: Isothermality (BIO 3), Temperature Seasonality (BIO 4), Minimum Temperature of Coldest Month (BIO 6), Temperature Annual Range (BIO 7), Annual Precipitation (BIO 12), Precipitation of Driest Month (BIO 14), Precipitation Seasonality (BIO 15), Precipitation of Warmest Quarter (BIO 18) and Precipitation of Coldest Quarter (BIO 19). We used seven algorithms to estimate population distribution, including generalized linear models (GLM), generalized additive models (GAM), multivariate adaptive regression splines (MARS), flexible discriminant analysis (FDA), general boosting method (GBM), random forests (RF) and maximum entropy (MaxEnt). In brief, 80 % of random samples from the species presence–absence data was used for model training and the remaining 20 % of data was used to evaluate model performance. Such random sampling was repeated 10 times and variable importance was evaluated by 100 permutations. After we calculated suitability for each single-ancestral population, suitability of each algorithm was standardized from 0 to 1 and weighted by their area under the curve (AUC) to obtain suitability mean. The variable importance was also weighted by the AUC after standardized. All computational works and figures were done by R packages ‘biomod2’ and R basic functions (R Core Team 2019; Thuiller *et al.* 2019).

### Niche characterization

To investigate niche differentiation among genetic groups, we performed multivariate analysis of variance (MANOVA), principal component analysis (PCA) and discriminant function analysis (DFA) after standardizing the nine bioclimatic variables. All these analyses were done by R packages factoextra, FactoMineR and MASS (Venables and Ripley 2002; Lê *et al.* 2008; Kassambara and Mundt 2017). Principal component analysis was done with the bioclimatic variables of northern population, central population and southern population altogether. To reveal the divergent process from the northern population to the southern population, DFA was done with two subsets, one with the bioclimatic variables of the northern population and the central population and the other with those of the central population and the southern population. The association between the axis of niche differentiation and bioclimatic variables was calculated from the correlation between DFA scores and the nine bioclimatic variables. In addition, we calculated Schoener’s *D* to assess the niche overlap between populations using ecospat.niche.overlap function in R package ecospat (Schoener 1968; Warren *et al.* 2008; Broennimann *et al*. 2012). The occurrence densities were created using the whole study area as background. Moreover, niche similarity was tested using ecospat.niche.similarity.test function in R package ecospat (Warren *et al.* 2008; Broennimann *et al*. 2012). The test was used the settings of alternative hypothesis as niche conservatism, 1000 replications and randomization of both tested populations.

### Association study of bioclimatic variables

To further investigate the association between bioclimatic variables and genetic variation, we used the nine bioclimatic variables (BIO 3, 4, 6, 7, 12, 14, 15, 18 and 19) to perform gradient forest analysis (Ellis *et al.* 2012). Gradient forest analysis identifies the association between genetic variation and environmental variables along with environmental gradients. Consequently, the most important environmental variables (i.e. the ones highly associated with genetic variation) and their response thresholds (i.e. the overall cumulative response to the environmental gradient) can be identified. Previous studies have revealed that underestimation of overall cumulative importance would occur when using less environment-associated SNPs in gradient forest analysis (Keller *et al.* 2018). Therefore, we performed genome-wide association study (GWAS) with kinship correction for the nine bioclimatic variables in TASSEL (Yu *et al.* 2006; Bradbury *et al.* 2007; Zhang *et al.* 2010). BIO 12, 14, 18 and 19 were transformed by natural log due to the skewed distributions. The top 200 candidate SNPs (about 1 percentile) were used for the downstream gradient forest analysis. Missing data were imputed using TASSEL Numerical Impute. Gradient forest analysis was performed with R package gradientForest (Ellis *et al.* 2012). For each value of an environment gradient, the gradient forest method estimates amount of allele frequency variation explained by this split and finally reports a cumulative *R*^2^ along the environmental gradient. One could therefore estimate the ‘genetic offset’ under future climate change: the change of *R*^2^ for the future versus current environments as an approximation of genetic changes required to be still adaptive under future climate change (Fitzpatrick and Keller 2015).

## Results

### *Solanum pimpinellifolium* diverged from Ecuador

We used the posterior probability of ADMIXTURE from Lin *et al.* (2019) to re-classify the genetic groups of 94 *S. pimpinellifolium* accessions. A total of three single-ancestral populations were assigned, including the northern population (8 accessions), the central population (25 accessions) and the southern population (21 accessions). The rest accessions were classified as the centre-north population (the 27 hybrid accessions of the northern population and the central population) and the centre-south population (the 13 hybrids of the central population and the southern population) **[see****Supporting Information—Table S1****]**. The geographic ranges of these populations showed a gradient along the west side of the Andes from north to south: the northern population occupied in Ecuador; the central population and the centre-north population were located in the north of Peru; the centre-south population lives in the middle of Peru and the southern population scattered in the south of Peru (Fig. 1). The phylogenetic tree indicated that the southern population was nested within the central population, which was further nested within the northern population (Fig. 1). The phylogeny is also consistent with amounts of polymorphism within each group: the northern population has the highest mean pairwise nucleotide polymorphism (π = 0.0019), followed by the central population (π = 0.0013) and the southern population (π = 0.0009). The results therefore suggested the northern population was the ancestral group and these genetic groups diverged southwards. The northern population was likely the ancestral population; later the southern subset of the northern population migrated to northern Peru and became the central population. Finally, the southern population was formed from the southern subset of the central population migrating further south (Fig. 1). Our results did not support previous ideas that northern Peru is the original centre of *S. pimpinellifolium* (Rick *et al.* 1977) but rather suggested genetic groups of this species serially diverged in a north to south pattern.

### Population differentiation may be driven by adaptation to local environments

**Figure 2.**
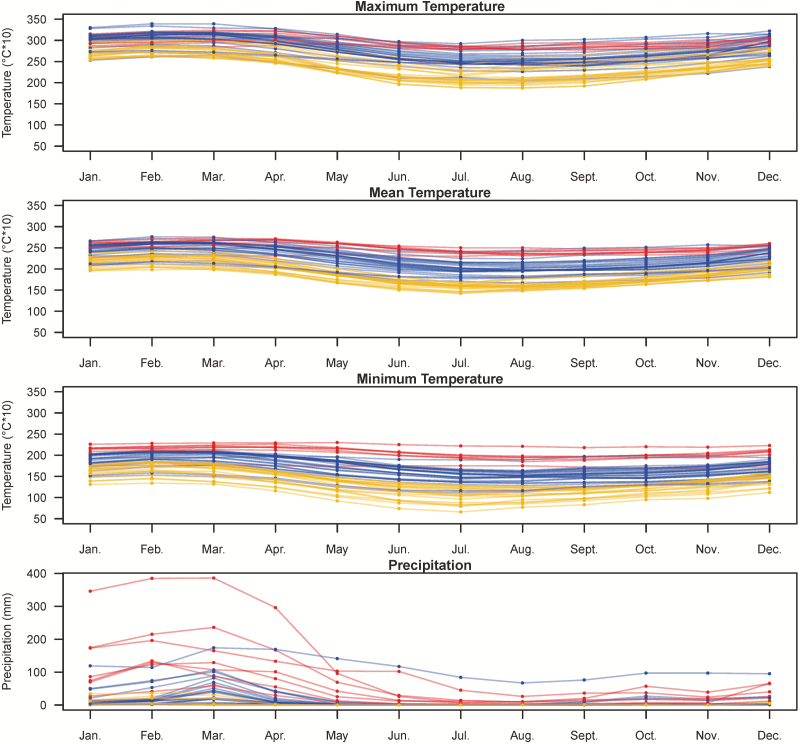
The temperature and precipitation per month of the collection site of each accession. The red curves indicate the northern population; blue for the central population; goldenrod for the southern population.

**Figure 3.**
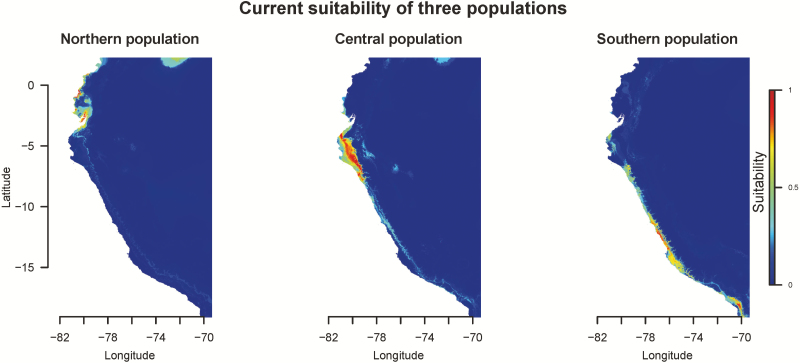
Species distribution modelling under current climatic condition.

To investigate whether the pattern of divergence is caused by purely neutral forces or likely environmental adaptation, we first investigated the habitats of these genetic groups. The northern population lived in the hottest environment while southern population lived in the coldest region (Fig. 2). The rainfall was concentrated during summer for the northern population and the central population while the southern population lived in a desert (Fig. 2). Besides, the habitat of the northern population has higher minimum temperature per month and more precipitation in summer than that of the central population (Fig. 2). We used nine bioclimatic variables, BIO 3, 4, 6, 7, 12, 14, 15, 18 and 19, to characterize the niches of these genetic groups by seven algorithms. The AUC of species distribution modelling ranged from 0.859 to 1, suggesting these seven models were distinguishable between presence and absence (Table 1). Therefore, we integrated these seven algorithms by weighted AUC to investigate the niches of these three populations. As a result, these three genetic groups had different niches. The northern population clustered at only a small coastal region in Ecuador, and its most important bioclimatic variable was minimum temperature of coldest month (Fig. 3; Table 2). The central population occupied northern Peru; temperature seasonality and annual precipitation showed almost equally contribution to its niche. The southern population clustered in southern Peru and its niche was contributed heavily by temperature seasonality and precipitation of warmest quarter.

**Table 1.** The AUC of the seven models of the three single-ancestral populations.

AUC	GLM	GAM	MARS	GBM	RF	FDA	MaxEnt
Northern population	0.997	1	0.997	0.999	1	0.868	0.991
Central population	0.991	1	0.991	1	1	0.932	0.859
Southern population	0.972	0.997	0.987	1	1	0.94	0.904

**Table 2.** The variable importance of the three single-ancestral populations.

Variable importance	Northern population	Central population	Southern population
BIO 3	0.0183	0.0283	0.0464
BIO 4	0.0235	0.1997	0.2898
BIO 6	0.2921	0.1693	0.0398
BIO 7	0.0298	0.0733	0.0545
BIO 12	0.1985	0.1882	0.1313
BIO 14	0.1415	0.0519	0.0359
BIO 15	0.1876	0.0516	0.0811
BIO 18	0.029	0.1452	0.2366
BIO 19	0.0585	0.0601	0.0561

**Figure 4.**
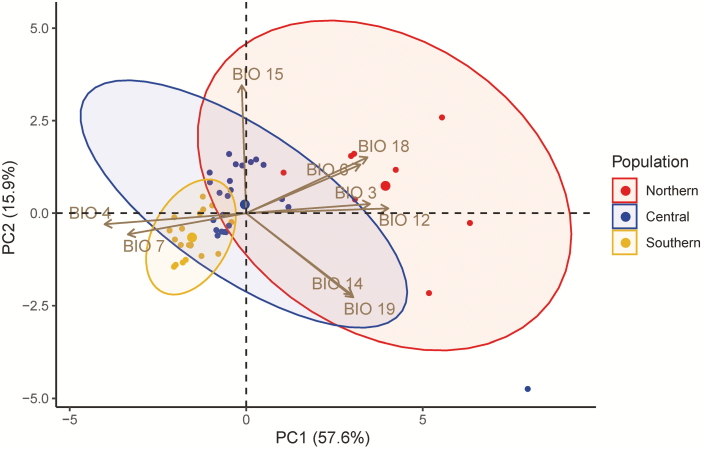
Principal component analysis of the nine bioclimatic variables. Ellipse means 95 % confidence interval. The BIO codes indicate Isothermality (BIO 3), Temperature Seasonality (BIO 4), Minimum Temperature of Coldest Month (BIO 6), Temperature Annual Range (BIO 7), Annual Precipitation (BIO 12), Precipitation of Driest Month (BIO 14), Precipitation Seasonality (BIO 15), Precipitation of Warmest Quarter (BIO 18) and Precipitation of Coldest Quarter (BIO 19).

Multivariate analysis of variance suggested that the niches of these genetic groups were different from each other (*P-*value < 0.001). Besides, niche similarity tests were not significant for the northern population and the central population (*P*-value = 0.35), the northern population and the southern population (*P*-value = 0.38) and the central population and the southern population (*P*-value = 0.11). We also used Schoener’s *D* to assess niche overlap, an index ranging from 0 (no niche overlap) to 1 (identical niches) (Schoener 1968; Warren *et al.* 2008). Schoener’s *D* were 0.01, 0.00 and 0.26 for the northern population and the central population, the northern population and the southern population, and the central population and the southern population, respectively. These results confirmed these population had different niche spaces. We further plotted PCA of the bioclimatic variables to visualize the difference among populations. PC1 and PC2 explained 57.6 % and 15.9 % variation (Fig. 4). The northern population had the largest niche space while the southern population had the smallest (Fig. 4), consistent with their genetic diversity and further implied that the northern population was the ancestral group even though it had the smallest sample size and geographical distribution. Besides, the divergence from the northern population to the southern population was generally in the direction of decreasing temperature and precipitation (BIO 6, 12 and 18) and increasing temperature variation (BIO 4 and 7) (Fig. 4; Table 3). The direction of divergence showed that as the genetic groups progressively diverged from north to south, they also differentiated in their niche space, and differential local adaptation contributed to the maintenance of their genetic divergence.

**Table 3.** The correlation between axes of niche divergence and bioclimatic variables.

BIO	Description	Northern population and central population	Central population and southern population
3	Isothermality	0.58	−0.09
4	Temperature Seasonality	−0.76	−0.35
6	Minimum Temperature of Coldest Month	0.82	0.85
7	Temperature Annual Range	−0.84	−0.78
12	Annual Precipitation	0.68	−0.18
14	Precipitation of Driest Month	0.14	0.09
15	Precipitation Seasonality	−0.14	0.05
18	Precipitation of Warmest Quarter	0.74	0.24
19	Precipitation of Coldest Quarter	0.35	0.10

### The spatial distribution of *S. pimpinellifolium* genetic variation is strongly affected by temperature

**Figure 5.**
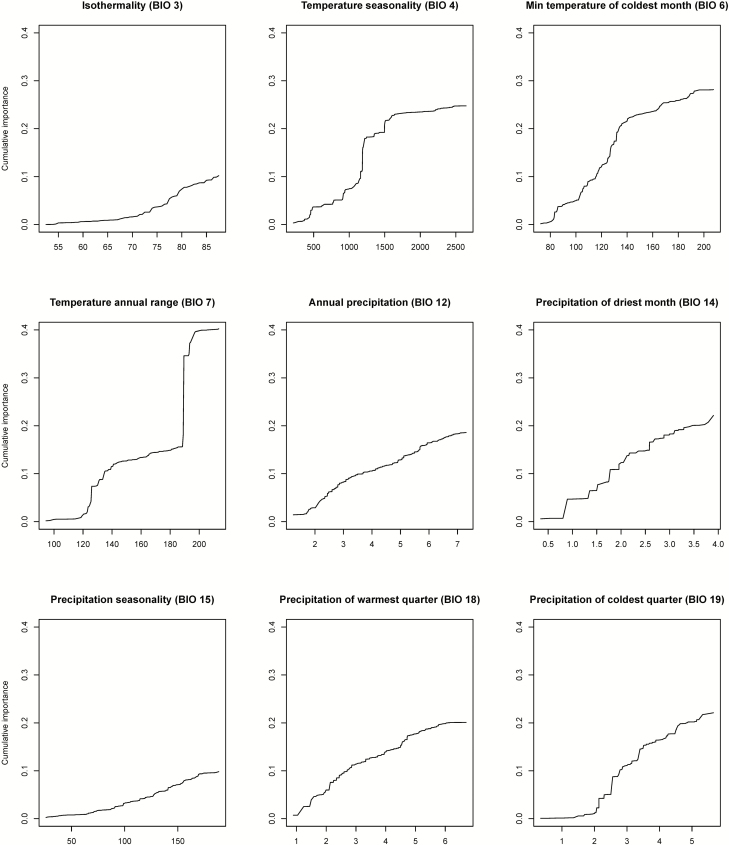
The overall cumulative importance of each bioclimatic variable from gradient forest. BIO 12, 14, 18 and 19 were done with natural log transform. The units of temperature and precipitation were °C * 10 and mm, respectively.

Top 200 GWAS SNPs associated with the nine bioclimatic variables were used to perform gradient forest analysis **[see****Supporting Information—Fig. S1****]**. Theoretically, if these bioclimatic variable-associated SNPs are linked with loci adaptive to the bioclimatic variables, a high overall cumulative importance can be observed (Fitzpatrick and Keller 2015; Keller *et al.* 2018). According to the overall *R*^2^-weighted cumulative importance, the most important three bioclimatic variables were annual temperature range, minimum temperature of coldest month and temperature seasonality (Fig. 5). This result indicated that temperature could be the primary factor to shape the genetic variation of *S. pimpinellifolium*. In addition, a dramatic increase of overall cumulative importance was observed for annual temperature range in a range from 12–14 °C to 18–20 °C, indicating two response thresholds for this bioclimatic variable (Fig. 5). Besides, the pattern of single-SNP importance also implied two clusters of potentially adaptive genes to temperature annual range: one cluster showing the rapid increase of overall cumulative importance at 12–14 °C and the other at 18–20 °C **[see****Supporting Information—Fig. S2****]**. Meanwhile, for temperature seasonality (standard derivation × 100), a sudden increase of overall cumulative importance was observed in a range from 1200 to 1300 (Fig. 5). These response thresholds suggested that some of these bioclimatic associated alleles could devote to the ranges of these bioclimatic variables together. Other bioclimatic associated alleles increased the overall cumulative importance progressively, implying these alleles might respond to the bioclimatic variables individually.

### Different populations face different fates under climatic change

**Figure 6.**
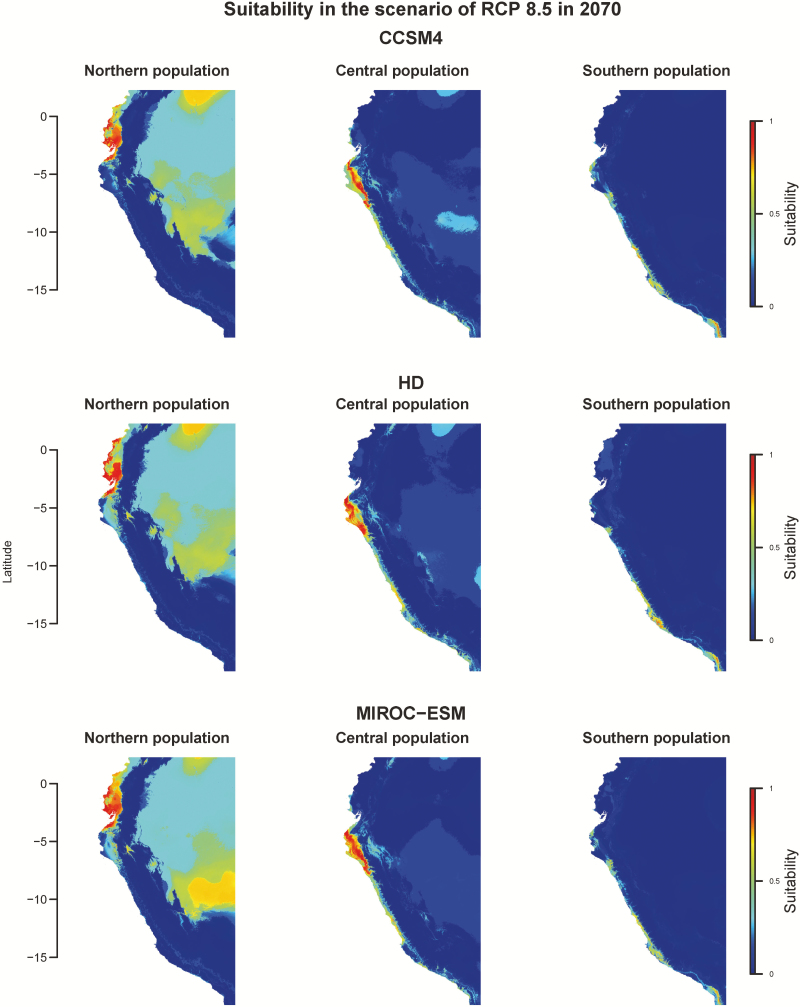
The projection of future species distribution on the scenario of RCP 8.5 in 2070.

**Figure 7.**
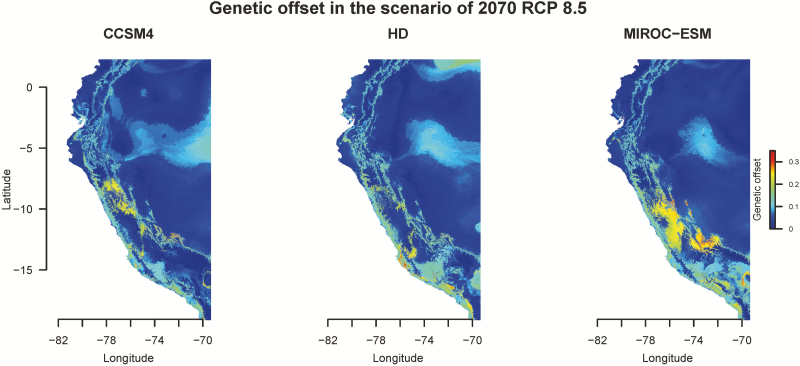
Genetic offset in a scenario of RCP 8.5 in 2070. High genetic offset (red) means the less association between genetic variation and bioclimatic variables than current environment, implying an extinction risk for the populations in these regions. Since the definition of genetic offset is the genetic mismatch of current populations to future climatic change, one should only focus on the results in geographic regions occupied by current populations (along the coast).

We have shown that *S. pimpinellifolium* is sensitive to temperature and precipitation, and genetic variation exists for differential adaptation to temperature-related variables. To investigate whether anthropogenic climate change will impact the future survival of *S. pimpinellifolium*, we predicted the species distribution in 2050 and 2070 using two approaches. The first one was to project the current bioclimatic variables on future bioclimatic variables and estimate suitability. In a scenario of RCP 8.5 in 2070, three global climatic models all suggested that the northern population would expand its own range when comparing to the current distribution, and the central population would maintain its current range (Fig. 6). In contrast to the optimistic future of the northern and central population, the southern population would reduce its spatial distribution (Fig. 6). In the rest scenarios (the combinations of RCP 2.6 and RCP 8.5 in 2050 and 2070), the future species distributions all presented similar trends with notable change of their habitats **[see****Supporting Information—Fig. S3****]**. The second approach was applying gradient forest analysis on the estimation of genetic offset (Fitzpatrick and Keller 2015), which approximates the magnitude of genetic change that a regional population must make to maintain the association between its genetic composition and its environment. In the scenario of RCP 8.5 in 2070, small genetic offset was revealed in the north and the centre of the current species range (Fig. 7). Meanwhile, the south of whole species range showed higher genetic offset (Fig. 7). The similar trend was observed in the scenario of RCP 8.5 in 2050 but less genetic offset was observed in the scenarios of RCP 2.6 **[see****Supporting Information—Fig. S4****]**. These projections suggested southern accessions would require certain genetic change to remain locally adaptive to warming environments if the greenhouse gas emission is not well controlled. Combining the results of the seven algorithms and gradient forest analysis, different fates were revealed among these three populations. The global warming would create a better environment for the northern, more tropical, population but reduce the distribution range for the southern, more temperate, population.

## Discussion

### *Solanum pimpinellifolium* adapted to cold and drought during its differentiation

Previous studies suggested that northern Peru should be the original centre of *S. pimpinellifolium* because accessions in this region contained the most allele numbers and presented the highest expected heterozygosity (Rick *et al.* 1977; Zuriaga *et al.* 2009, Blanca *et al.* 2015; Lin *et al.* 2019). Without the support of phylogenetic analysis, higher genetic variation could potentially result from admixture of the northern population and the central population, given that some accessions could outcross **[see****Supporting Information—Table S1****]**. Besides, high outcrossing rate could spontaneously maintain the genetic diversity of these accessions in northern Peru (Rick *et al.* 1977). However, in this study, we propose an alternative hypothesis that *S. pimpinellifolium* was originated from Ecuador (the northern population) and subsequently diverged southwards into the central population and the southern population. The northern population occupies the basal position of the phylogenetic tree and shows the highest genetic diversity when comparing among the three non-admixed populations. In addition, the northern population occupies the largest niche space despite its relatively small geographic range, consistent with its high genetic variation (Fig. 4). Moreover, the genetic divergence matched a progressive decrease of temperature and precipitation in the niche space, suggesting *S. pimpinellifolium* became more adjustable to cold and drought when it migrated southwards. This hypothesis brings out other questions. To our knowledge, *S. pimpinellifolium* ranges only from Ecuador to Peru, and only on the west side of the Andes (Moyle 2008; Tomato Genetics Resource Center, UC Davis). The landscape limits the west-east expansion of *S. pimpinellifolium* naturally but what limits its northward expansion. If the northern population is really more adaptive to warmer or more humid environments, could we collect any sample in Colombia? If none of any *S. pimpinellifolium* could be found in Colombia, why not? These questions deserve further investigation.

### Species distribution modelling with and without genetic variation reported different sets of climatic variables

In this study, we identified the important bioclimatic variables of the niches of these three genetic groups by species distribution modelling with and without genetic variation. Although these two approaches resulted in similar species distribution predictions in the future, the important climatic variables were reported differently. Without the genetic variation, species distribution modelling suggested both temperature- and precipitation-related variables were the most important factors that determine the species distribution of *S. pimpinellifolium.* Accounting for intraspecific genetic variation, our gradient forest analysis reveals a more relevant role of temperature-related variables (Fig. 5). Algorithms not considering genetic variation predict suitability of a species by comparing presence locations to background/absence in an assumption that individuals of a species respond to environment equally, ignoring genetic variation and differential local adaptation within species (Phillips *et al.* 2006; Phillips and Dudík 2008; Fithian and Hastie 2013). However, results from these algorithms should be carefully interpreted since populations of a species have been revealed to respond differently to climate (Chardon *et al.* 2020). In this regard, gradient forest analyses by identifying the association between genetic variation and environmental variables within a species may provide more reliable predictions (Ellis *et al.* 2012). In our study system, gradient forest analysis reveals that temperature is the most important factor affecting differential local adaptation of *S. pimpinellifolium* populations. This result is supported by the evidence that the divergence from the northern population to the central population is highly correlated with temperature annual range, while that from the central population to the southern population is minimum temperature of coldest month (Table 3). Nevertheless, the genome–environment association would require more evidence to confirm that the local adaptation of *S. pimpinellifolium* is associated with temperature, and the association may also reflect another underlying environmental factor not used in our study, such as the length of growing seasons.

### Predictions of future species distribution are consistent with the niche features

When we used presence–background data and geographic information to predict the species distribution, the future species distributions of the northern population showed an expansion to most of western Ecuador while the southern population shrank its current habitat (Fig. 6). A similar future was revealed when species distribution was predicted by the incorporation of environmental information and genetic variation. In this approach, the northern population maintained its suitability while the southern population showed less adaptive to the future environment (Fig. 7). These results implied that the three populations of *S. pimpinellifolium* would have different fates under global warming conditions. According to our results, *S. pimpinellifolium* diverged from warmer to colder environments (Figs 1 and 4), indicating the southern population might lose heat-tolerant alleles. Regarding the narrow niche space of the southern population (Fig. 4), either the southern population would adjust the response to global warming conditions through phenotypic plasticity or it could be less adaptive to warming environment (Gienapp *et al.* 2008). The northern population, being the diverse ancestor and having the largest niche space, may be able to maintain adaptive alleles to different temperature range, providing it an opportunity to adapt to global warming conditions and/or extend its range (Fig. 4).

The fate of the northern population supports that populations with high genetic variation would have more ability to cope with climatic change (Theodoridis *et al.* 2018; Lai *et al.* 2019). However, while evolution towards a new adaptation optimum could certainly occur through the change of allele frequency of standing variation, such process of directional selection inevitably involves the death or lowered reproductive output of individuals bearing the non-adaptive alleles. Genetic variation would be greatly reduced during this process, especially when the process is rapid and allows little time for recombination, resulting in a genome-wide loss of variation. In the long run, such process is still detrimental for the species even though one could observe rapid adaptation. Therefore, high genetic diversity should be treated as a buffer that reduces the instant impact under changing environment, but this does not guarantee the future fate of a population.

## Supporting Information

The following additional information is available in the online version of this article—

**Figure S1.** Manhattan plots of GWAS.

**Figure S2.** The single-SNP cumulative importance of each bioclimatic variable in gradient forest.

**Figure S3.** The projections of species distribution on different scenarios in 2050 and 2070.

**Figure S4.** Genetic offset in different scenarios.

**Table S1.** The geographic information, mating system and the posterior probability of ADMIXTURE of 94 *S. pimpinellifolium* accessions (Lin *et al.* 2019).

plaa012_suppl_Supplementary_Data
