# Supplementary material for: The climatic association of population divergence and future extinction risk of Solanum pimpinellifolium
Source: AoB Plants. 2020 Mar 12;12(2):plaa012. doi: 10.1093/aobpla/plaa012 (PMC7107907; doi:10.1093/aobpla/plaa012)
Supplement: plaa012_suppl_Supplementary_Data [file plaa012_suppl_supplementary_data.pdf]

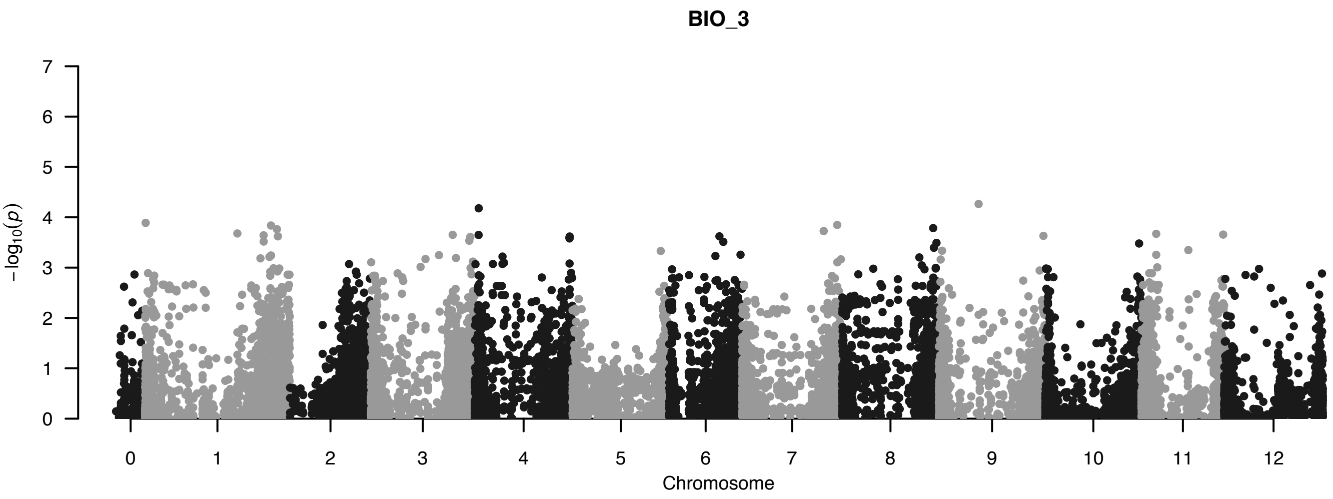

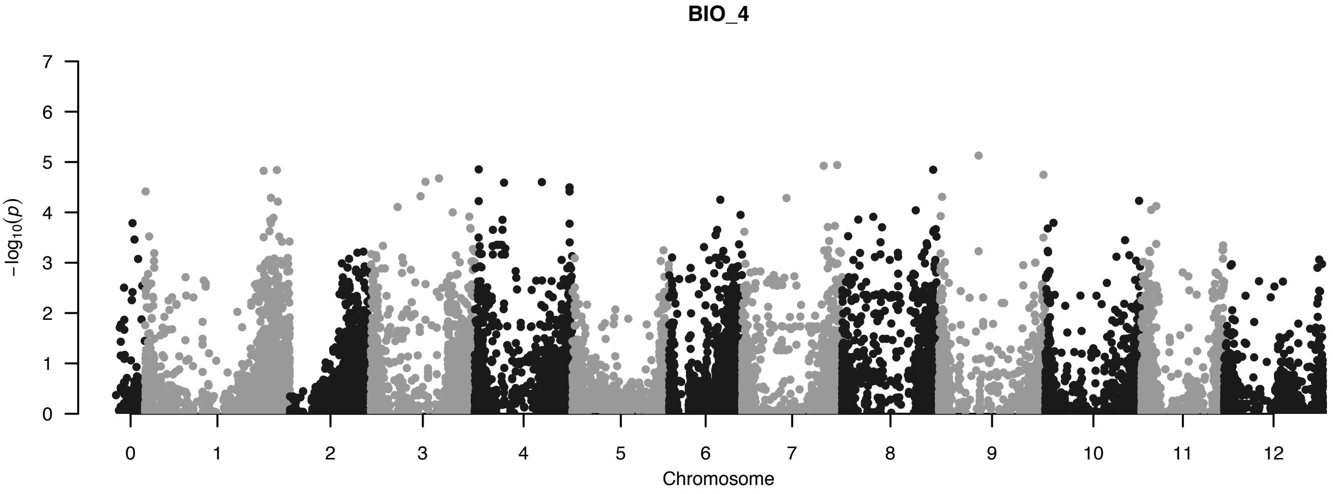

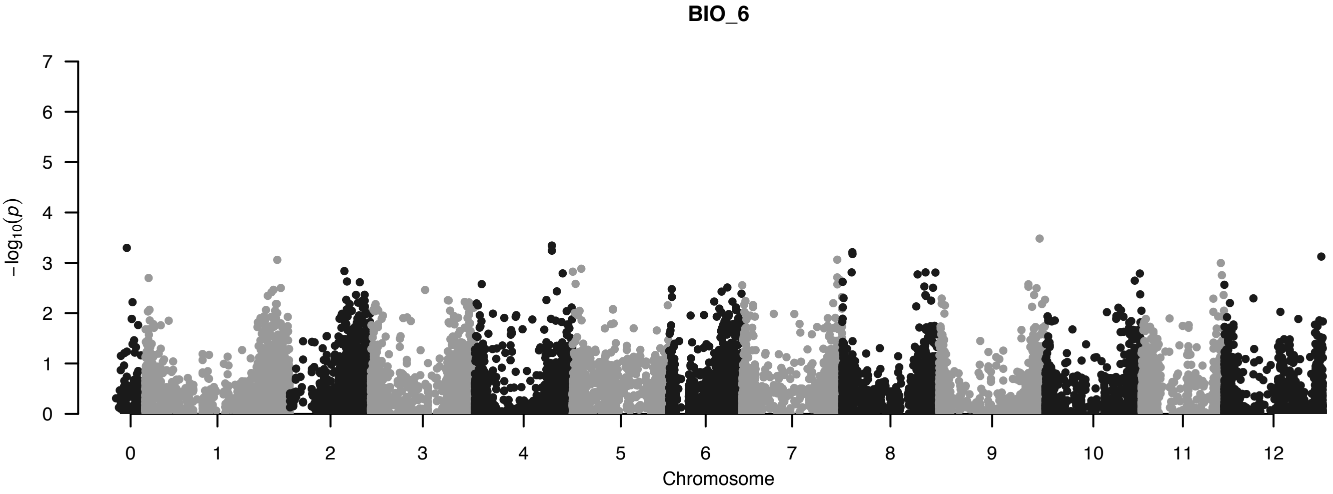

Figure S1 (page 1/3)

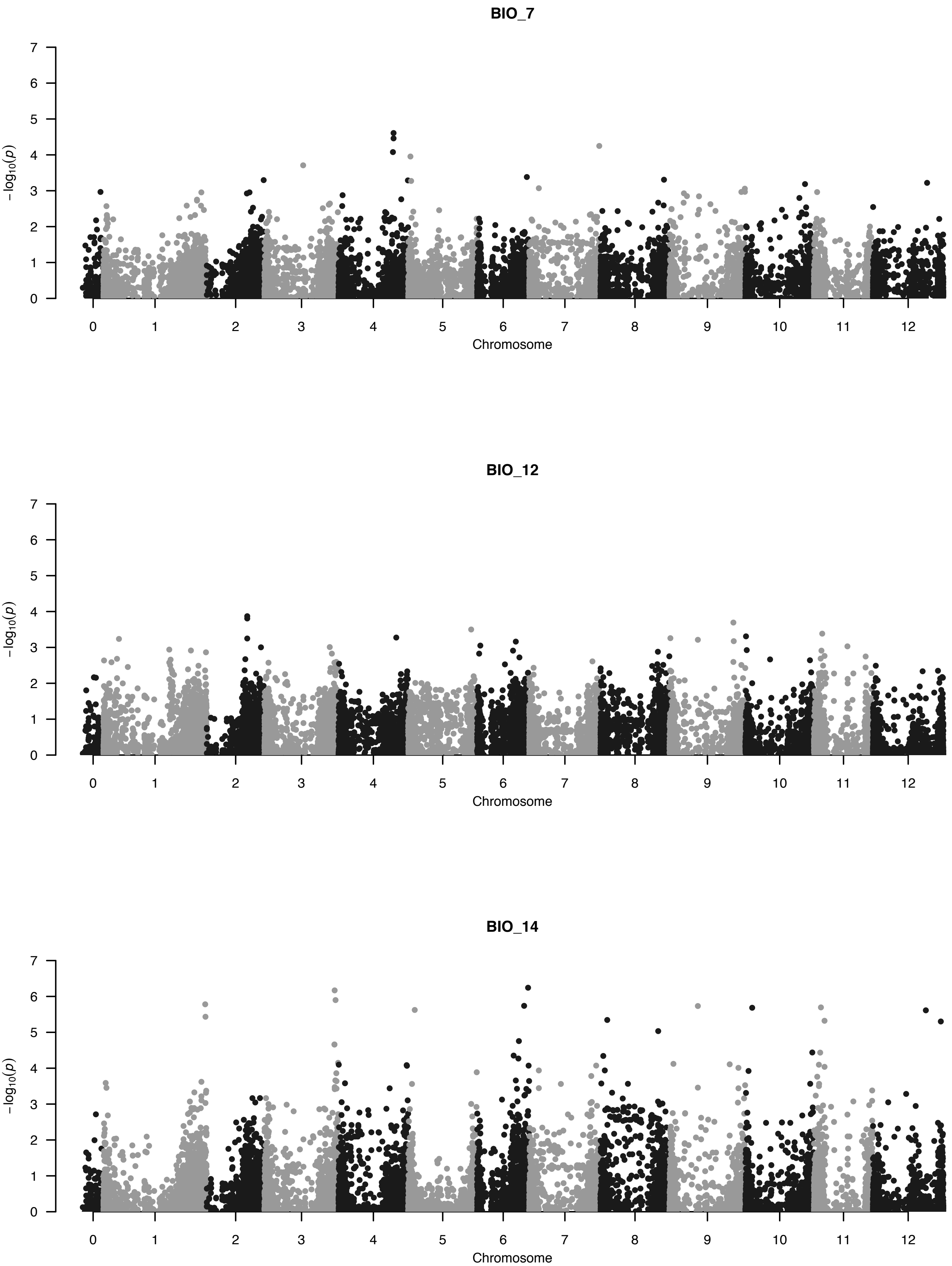

Figure S1 (page 2/3)

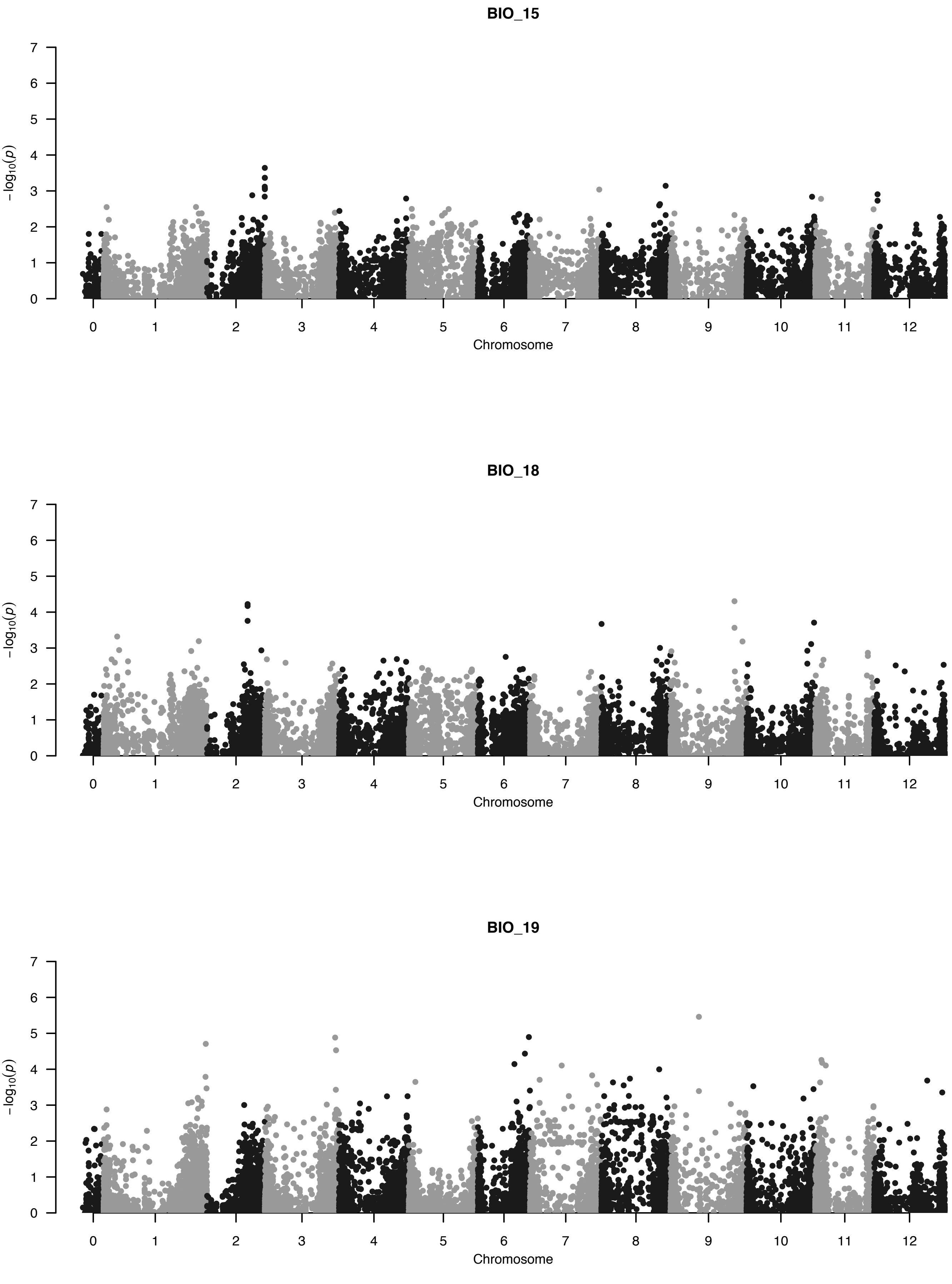

Figure S1 Manhattan plots of GWAS.

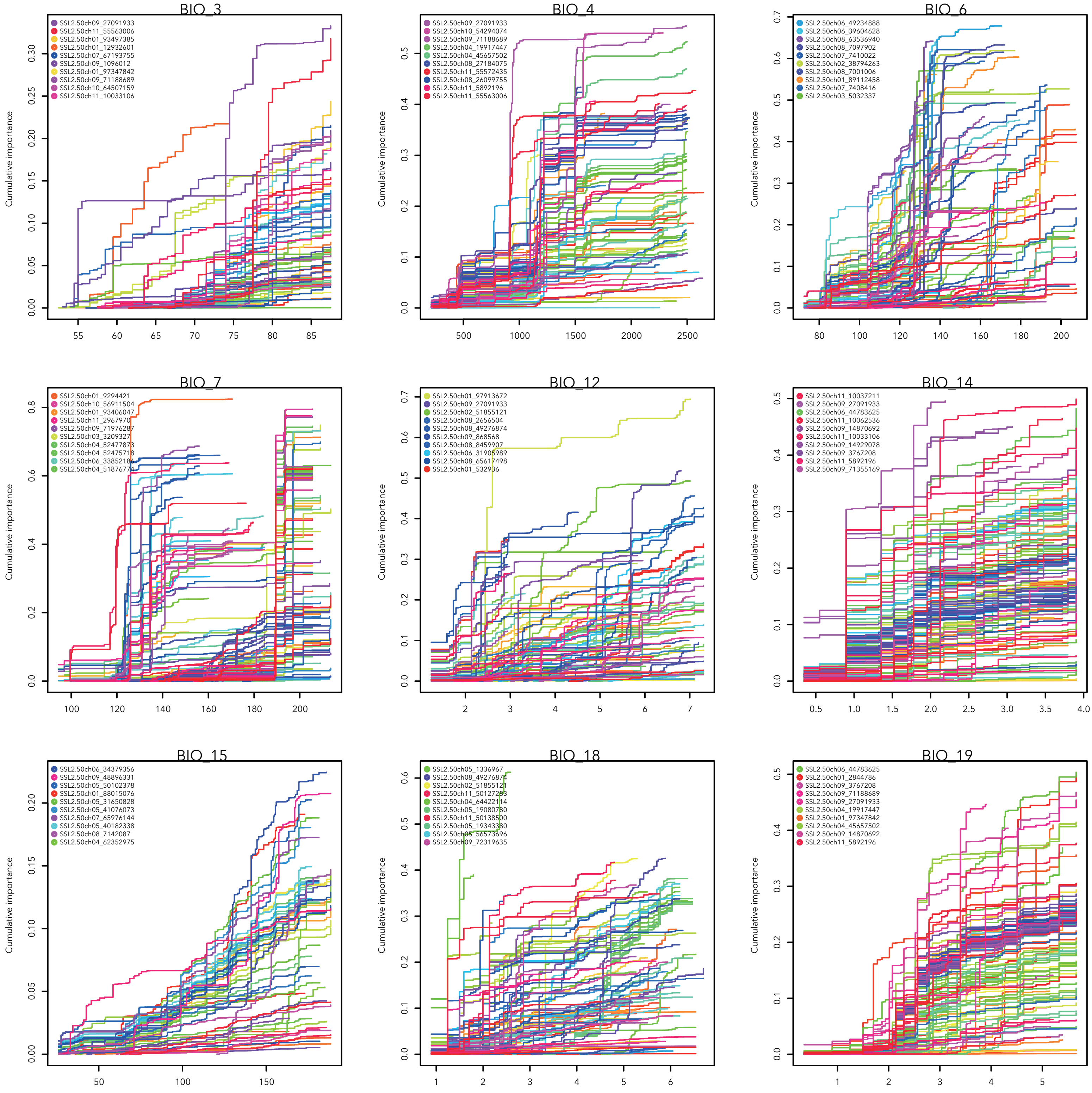

Figure S2 The single-SNP cumulative importance of each bioclimatic variable in gradient forest. The legend lists the top ten most important SNP of each bioclimatic variable. The units of temperature and precipitation were °C*10 and mm, respectively.

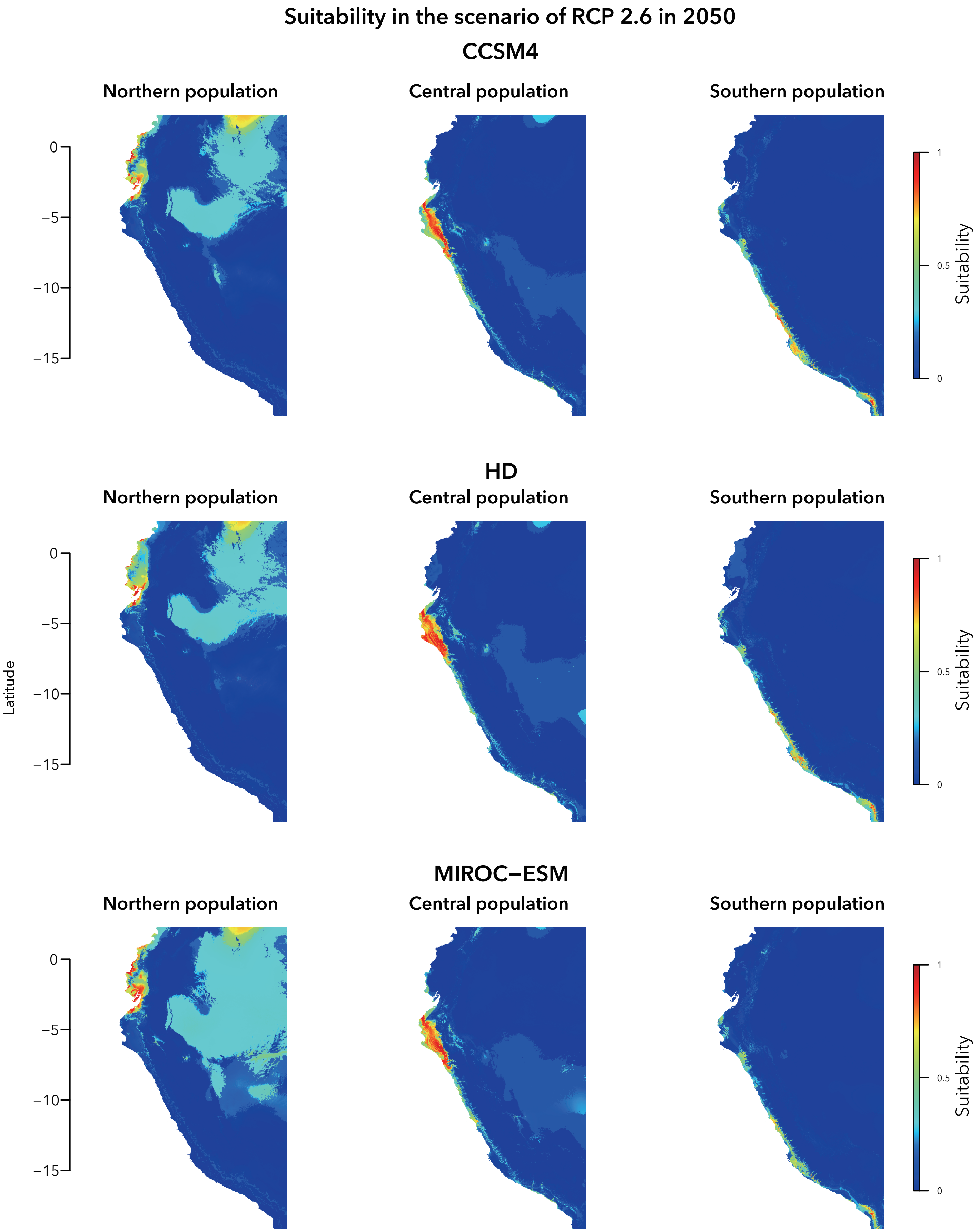

Figure S3 (page 1/3)

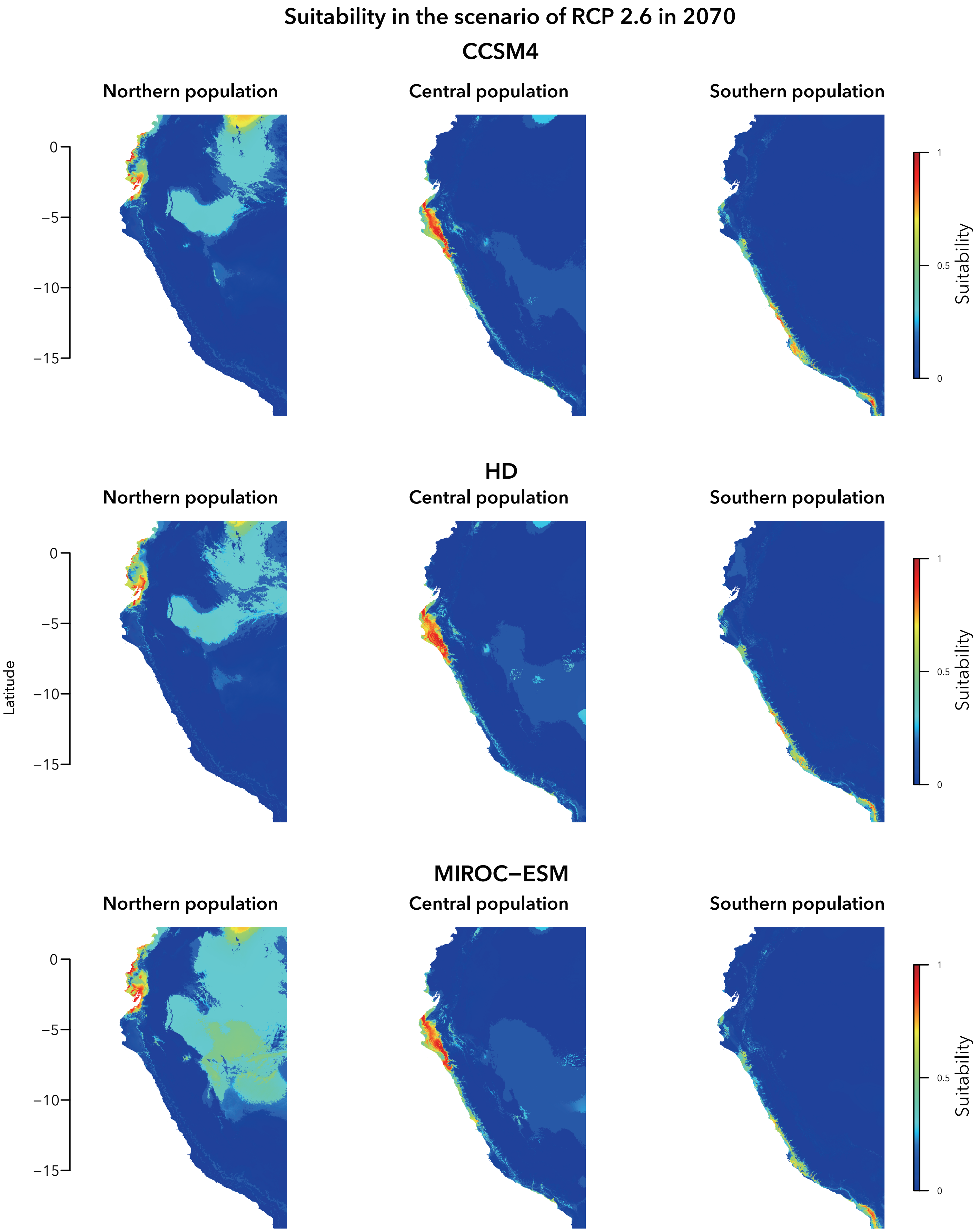

Figure S3 (page 2/3)

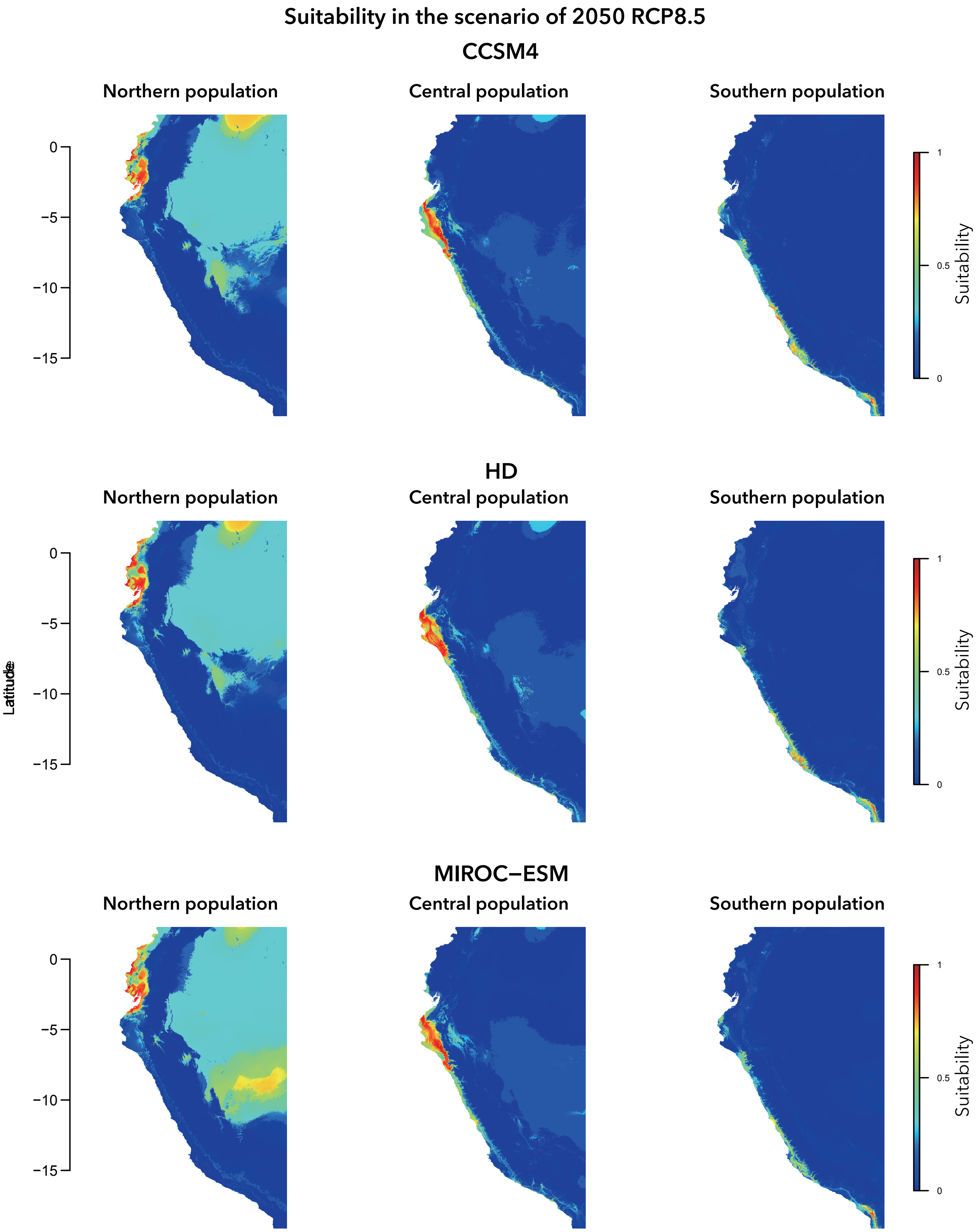

Figure S3 The projections of species distribution on different scenarios in 2050 and 2070.

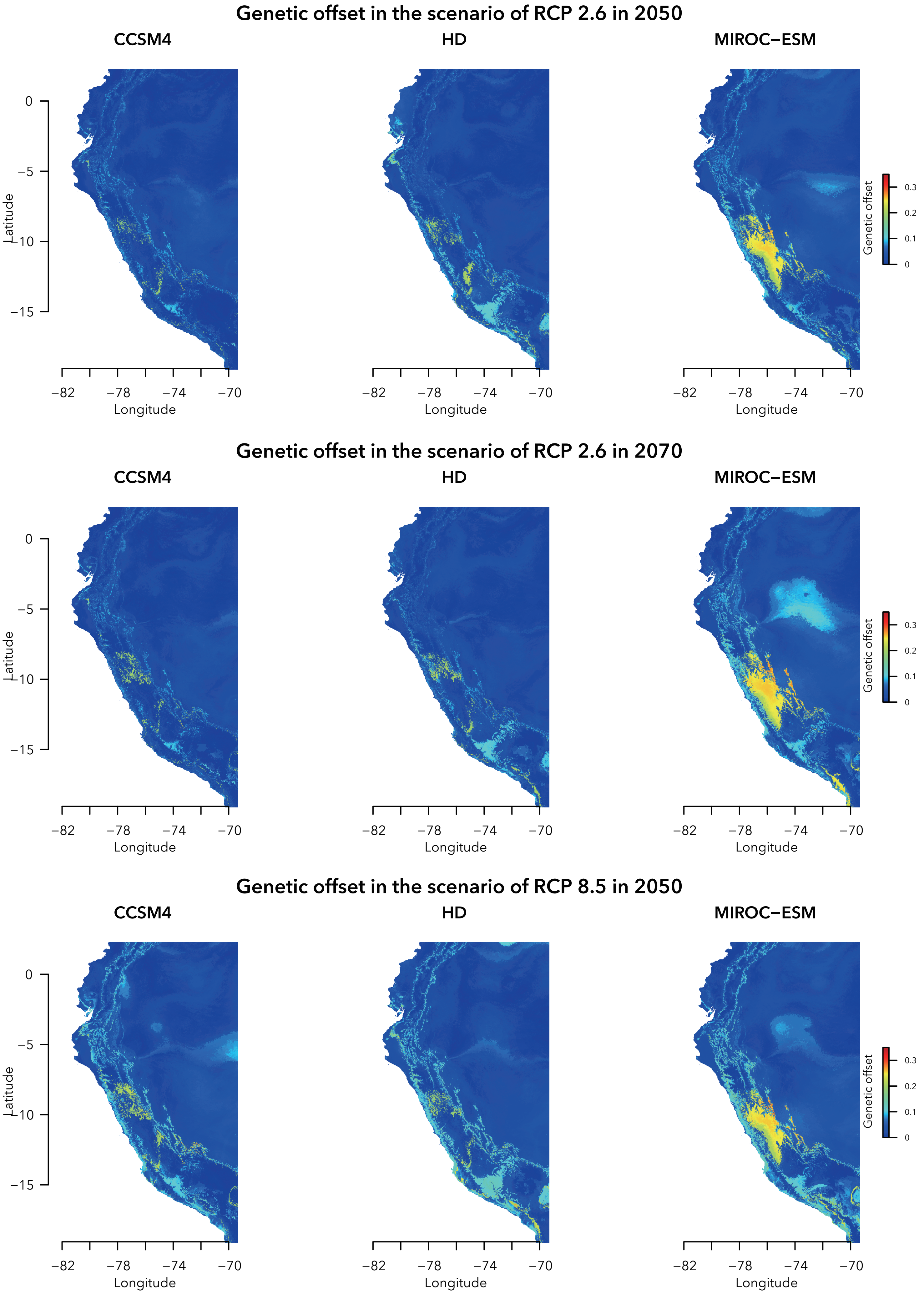

Figure S4 Genetic offset in different scenarios.

Table S1 The geographic information, mating system and the posterior probability of ADMIXTURE of 94 *S. pimpinellifolium* accessions (Lin *et al.*, 2019)

ID	POP	Longitude	Latitude	Mating.system	Q3_1	Q3_2	Q3_3
LA0114	Central population	-79.5667	-7.4	FSC	1.00E-05	1.00E-05	0.99998
LA0373	Center-south population	-78.23	-9.94	ASC	1.00E-05	0.468622	0.531368
LA0391	Central population	-78.6817	-7.2442	ASC	1.00E-05	1.00E-05	0.99998
LA0397	Center-north population	-79.7167	-6.75	FSC	0.195544	1.00E-05	0.804446
LA0400	Center-north population	-79.9642	-5.2608	FSC	0.221763	1.00E-05	0.778227
LA0417	Northern population	-79.9167	-2.7333	ASC	0.99998	1.00E-05	1.00E-05
LA0442	Center-south population	-78.2592	-9.4817	FSC	1.00E-05	0.389707	0.610283
LA1236	Center-north population	-79.15	-0.25	ASC	0.221955	0.067422	0.710623
LA1237	Northern population	-79.85	0.8667	ASC	0.99998	1.00E-05	1.00E-05
LA1245	Northern population	-79.9667	-3.4583	ASC	0.99998	1.00E-05	1.00E-05
LA1246	Northern population	-79.36	-3.99	ASC	0.903568	1.00E-05	0.096422
LA1256	Northern population	-79.6167	-2.6667	ASC	0.99998	1.00E-05	1.00E-05
LA1261	Northern population	-79.5167	-1.8167	ASC	0.99998	1.00E-05	1.00E-05
LA1301	Southern population	-75.9167	-13.7333	ASC	1.00E-05	0.99998	1.00E-05
LA1335	Center-south population	-73.25	-16.4	ASC	1.00E-05	0.441659	0.558331
LA1348	Central population	-79.5	-7.45	FSC	1.00E-05	1.00E-05	0.99998
LA1349	Central population	-79.4997	-6.7436	ASC	1.00E-05	1.00E-05	0.99998
LA1371	Southern population	-76.6539	-11.8894	ASC	1.00E-05	0.99998	1.00E-05
LA1381	Center-north population	-79.9667	-5.5667	ASC	0.239473	1.00E-05	0.760517
LA1382	Center-north population	-78.0293	-6.8449	FSC	0.295675	1.00E-05	0.704315
LA1466	Center-south population	-79.3833	-6.6333	FSC	1.00E-05	0.48882	0.51117
LA1469	Center-north population	-79.79	-5.86	ASC	0.389416	0.006906	0.603678
LA1471	Central population	-79.75	-6.3167	FSC	0.026448	1.00E-05	0.973542
LA1478	Central population	-80.0833	-5.2167	FSC	0.048139	0.039619	0.912243
LA1514	Southern population	-77.1189	-11.0453	ASC	1.00E-05	0.99998	1.00E-05
LA1521	Southern population	-76.5053	-12.7647	ASC	1.00E-05	0.99998	1.00E-05
LA1547	Southern population	-77.9333	0.5833	ASC	1.00E-05	0.99998	1.00E-05
LA1576	Southern population	-76.8667	-12.1667	ASC	1.00E-05	0.99998	1.00E-05
LA1577	Central population	-79.18	-7.81	FSC	1.00E-05	0.083148	0.916842
LA1578	Central population	-79.5833	-7.3333	FSC	1.00E-05	1.00E-05	0.99998

LA1579	Central population	-79.87	-6.59	FSC	0.027768	1.00E-05	0.972222
LA1580	Central population	-79.87	-6.59	FSC	0.013032	1.00E-05	0.986958
LA1581	Central population	-79.89	-6.6	FSC	0.012581	1.00E-05	0.987409
LA1582	Central population	-79.7333	-6.15	FSC	0.007111	1.00E-05	0.992879
LA1583	Central population	-79.72	-6.23	FSC	0.030361	1.00E-05	0.969629
LA1584	Central population	-79.79	-6.37	FSC	0.028557	1.00E-05	0.971433
LA1585	Southern population	-79.4664	-6.6922	FSC	1.00E-05	0.99998	1.00E-05
LA1586	Central population	-78.73	-8.36	FSC	1.00E-05	1.00E-05	0.99998
LA1587	Central population	-79.5167	-7.4333	FSC	1.00E-05	1.00E-05	0.99998
LA1589	Center-south population	-78.74	-8.39	ASC	1.00E-05	0.124707	0.875283
LA1590	Center-south population	-78.73	-8.37	FSC	1.00E-05	0.109144	0.890846
LA1591	Central population	-79.1167	-7.7167	FSC	1.00E-05	0.07646	0.92353
LA1593	Center-south population	-78.67	-8.54	ASC	1.00E-05	0.162811	0.837179
LA1595	Center-south population	-78.47	-9.27	ASC	1.00E-05	0.701738	0.298252
LA1596	Center-south population	-78.5667	-8.925	FSC	1.00E-05	0.226335	0.773655
LA1599	Center-south population	-78.1833	-10.0583	ASC	1.00E-05	0.49316	0.50683
LA1601	Southern population	-77.68	-10.67	ASC	1.00E-05	0.99998	1.00E-05
LA1602	Southern population	-76.9833	-11.7833	ASC	1.00E-05	0.99998	1.00E-05
LA1606	Southern population	-76.2	-13.4667	FSC	1.00E-05	0.99998	1.00E-05
LA1615	Center-north population	-80.6333	-5.2333	ASC	0.305126	1.00E-05	0.694864
LA1617	Northern population	-80.4667	-3.5667	FSC	0.99998	1.00E-05	1.00E-05
LA1628	Central population	-79.55	-7.1667	ASC	1.00E-05	1.00E-05	0.99998
LA1629	Southern population	-77.0333	-12.1167	ASC	1.00E-05	0.99998	1.00E-05
LA1645	Southern population	-77.0333	-12.1314	ASC	1.00E-05	0.99998	1.00E-05
LA1659	Center-south population	-77.8586	-9.5467	ASC	1.00E-05	0.337606	0.662384
LA1670	Southern population	-70.5167	-17.8333	ASC	1.00E-05	0.99998	1.00E-05
LA1683	Center-north population	-81.11	-4.87	FSC	0.581789	1.00E-05	0.418201
LA1684	Center-north population	-80.15	-5.1	FSC	0.317832	1.00E-05	0.682158
LA1685	Center-north population	-80.6975	-4.8867	FSC	0.520265	1.00E-05	0.479725
LA1686	Center-north population	-80.62	-5.07	FSC	0.331952	1.00E-05	0.668038
LA1687	Center-north population	-80.62	-5.07	FSC	0.135376	1.00E-05	0.864614
LA1688	Center-north population	-80.375	-4.8833	FSC	0.365268	1.00E-05	0.634722

LA1689	Center-north population	-80.6175	-5.1764	FSC	0.595418	1.00E-05	0.404572
LA1690	Center-north population	-80.6175	-5.1764	FSC	0.335564	1.00E-05	0.664426
LA1720	Center-south population	-78	-9.5167	ASC	1.00E-05	0.34506	0.65493
LA1729	Southern population	-75.6406	-13.2969	ASC	1.00E-05	0.99998	1.00E-05
LA1921	Southern population	-75.1272	-14.3119	ASC	1.00E-05	0.99998	1.00E-05
LA1923	Southern population	-75.2833	-14.6667	ASC	1.00E-05	0.99998	1.00E-05
LA1924	Southern population	-75.2142	-14.6289	ASC	1.00E-05	0.99998	1.00E-05
LA1933	Southern population	-74.4458	-15.4564	ASC	1.00E-05	0.99998	1.00E-05
LA1936	Southern population	-74.0325	-15.8336	ASC	1.00E-05	0.99998	1.00E-05
LA2097	Center-north population	-79.9181	-4.3939	ASC	0.733049	1.00E-05	0.266941
LA2102	Center-north population	-79.4675	-4.4017	ASC	0.277929	1.00E-05	0.722061
LA2146	Central population	-79.4161	-7.3019	ASC	1.00E-05	1.00E-05	0.99998
LA2149	Central population	-78.7878	-7.2181	ASC	1.00E-05	1.00E-05	0.99998
LA2173	Central population	-78.7905	-5.3307	ASC	1.00E-05	1.00E-05	0.99998
LA2181	Center-north population	-78.7831	-5.7758	ASC	0.319561	1.00E-05	0.680429
LA2183	Center-north population	-78.67	-5.74	ASC	0.708783	1.00E-05	0.291207
LA2186	Center-north population	-78.1667	-5.8917	ASC	0.816527	1.00E-05	0.183463
LA2389	Central population	-79.1333	-7.25	FSC	1.00E-05	1.00E-05	0.99998
LA2390	Central population	-79.1417	-7.2333	ASC	1.00E-05	1.00E-05	0.99998
LA2401	Center-south population	-78.2278	-9.5083	ASC	1.00E-05	0.368292	0.631698
LA2533	Southern population	-77.36	-11.3	ASC	1.00E-05	0.99998	1.00E-05
LA2645	Center-north population	-80.1833	-5.1667	FSC	0.307913	1.00E-05	0.692077
LA2646	Center-north population	-79.8	-5.05	FSC	0.37607	1.00E-05	0.62392
LA2647	Center-north population	-79.9833	-5.175	FSC	0.30155	1.00E-05	0.69844
LA2652	Center-north population	-80.6842	-4.9031	FSC	0.33139	1.00E-05	0.6686
LA2653	Center-north population	-80.5833	-4.75	FSC	0.525183	1.00E-05	0.474807
LA2655	Center-north population	-80.825	-4.9083	ASC	0.525661	1.00E-05	0.474329
LA2656	Center-north population	-80.7	-3.8	FSC	0.551157	1.00E-05	0.448833
LA2659	Central population	-80.625	-5.2167	FSC	1.00E-05	1.00E-05	0.99998
LA2852	Northern population	-80.4833	-0.8333	ASC	0.99998	1.00E-05	1.00E-05
LA2915	Central population	-79.7453	-5.9847	FSC	1.00E-05	1.00E-05	0.99998
LA3638	Southern population	-76.3167	-12.5667	ASC	1.00E-05	0.99998	1.00E-05
